# Ethyl Pyruvate Promotes Wound Healing in Elastase-Induced Lung Injury in Mice as Assessed by Hyperpolarized ^129^Xe Magnetic Resonance Imaging

**DOI:** 10.1007/s11307-025-02073-6

**Published:** 2025-12-10

**Authors:** Atsuomi Kimura, Akihiro Shimokawa, Neil J. Stewart, Rie Hosoi, Hirohiko Imai, Hideaki Fujiwara

**Affiliations:** 1 Division of Health Sciences, Graduate School of Medicine, The University of Osaka, 1-7 Yamadaoka, Suita, Osaka 565-0871 Japan; 2 Division of Clinical Medicine, School of Medicine & Population Health, Faculty of Health, POLARIS, University of Sheffield, Sheffield, UK; 3 Innovation Research Center for Quantum Medicine, Gifu University School of Medicine, 1-1 Yanagido, Gifu, 501-1194 Japan

**Keywords:** Hyperpolarized ^129^Xe magnetic resonance imaging, Elastase-induced lung injury, Mitogen-activated protein kinase, Ethyl pyruvate, Nicorandil

## Abstract

**Purpose:**

Wound healing process in lung injury involves the activation of the mitogen-activated protein kinase (MAPK) pathway. In this study, we investigated the role of the MAPK pathway in wound healing in a murine model of emphysema using hyperpolarized ^129^Xe (HP ^129^Xe) magnetic resonance imaging (MRI).

**Procedures:**

Porcine pancreatic elastase was administered intratracheally to 25 mice to induce lung injury. Temporal changes in pulmonary gas exchange function were monitored using HP ^129^Xe MRI, revealing a significant decline in function one day after elastase administration. Treatments with ethyl pyruvate (EP) and nicorandil (Nic), which upregulate and downregulate the MAPK pathway, respectively, were initiated in 12 and 7 of the 25 mice, respectively, and continued for 20 days. Over the 21-day period, HP ^129^Xe MRI was performed to monitor the disease progression and treatment efficacy through changes in the metrics of gas exchange and fractional ventilation.

**Results:**

HP ^129^Xe MRI showed that EP significantly improved gas exchange function 14 days after elastase administration, whereas Nic did not show any improvement. Ventilatory function also improved in the EP group, but not in the Nic group, 14 days after elastase administration. Histological analysis showed that EP repaired tissue damage to a level similar to that observed in healthy mice, whereas Nic did not.

**Conclusions:**

In the present study, we provide some insight into the role of the MAPK pathway in wound healing in elastase-induced lung injury, as assessed using the HP ^129^Xe MRI protocol.

**Supplementary Information:**

The online version contains supplementary material available at 10.1007/s11307-025-02073-6.

## Introduction

Hyperpolarized ^129^Xe (HP ^129^Xe) magnetic resonance imaging (MRI) is an established tool for clinically imaging pulmonary function [1–3]. This method has been applied for the functional assessment of various lung diseases in humans [4]. In a previous study, we developed a unique continuous-flow type HP ^129^Xe polarizer and applied it to murine pulmonary functional imaging under spontaneous breathing, which allowed us to evaluate pulmonary function under natural conditions [5, 6]. Recently, we have utilized our HP ^129^Xe MRI preclinical evaluation system to identify therapeutic drugs for several lung diseases [7–10]. Ethyl pyruvate (EP) was shown to be effective in repairing lung tissue and improving pulmonary function damaged by diseases such as chronic obstructive pulmonary disease (COPD), pulmonary fibrosis, and lung cancer [8–10].

A hallmark of lung diseases is the involvement of high mobility group box 1 (HMGB1), a damage-associated molecular pattern molecule, in disease progression [11–14]. Upon lung injury, HMGB1 is released from macrophages and cells undergoing apoptosis or necrosis, which binds to Toll-like receptor 4 (TLR4) and the receptor for advanced glycation end products (RAGE), which are highly expressed in alveolar epithelial cells [14, 15]. After binding to TLR4, HMGB1 activates the transcription factor nuclear factor kappa B (NF-κB) and induces inflammatory responses [16]. Furthermore, after binding to RAGE, HMGB1 generates reactive oxygen species (ROS) in the cytoplasm [17], and these ROS activate the mitogen-activated protein kinase (MAPK) signaling pathway in addition to the NF-κB pathway, exacerbating the pathology [18, 19].

In contrast, moderate activation of extracellular signal-regulated kinases (ERK) (1/2), which are involved in the MAPK pathway, has been reported to be paradoxically involved in wound healing in skin disorders and diabetes-induced tissue damage [20–22]. Overexpression of HMGB1 has been shown to aggravate the pathology of lung diseases [15, 23], whereas EP has been reported to downregulate HMGB1 production, deactivate NF-κB, and upregulate the expression of ERK(1/2) in a dose-dependent manner [24–26]. EP has also been reported to increase intracellular ROS and activate ERK to inhibit melanogenesis in B16F10 melanoma cells for the treatment of hyperpigmentation disorders [27].

Here, we hypothesize that EP modulates the expression levels of HMGB1 and intracellular ROS, deactivate NF-κB, and moderately activates the MAPK/ERK pathway to exert coordinated anti-inflammatory and tissue repair effects and improve pulmonary function. In this study, we attempted to confirm this hypothesis by observing the treatment response to EP in elastase-induced acute lung injury using HP ^129^Xe MRI. To the best of our knowledge, there have been no reports on the application of EP in the treatment of elastase-induced lung injury. The treatment response of EP was compared with that of nicorandil (Nic), which exhibits its protective effect against lung injury through suppression of ROS production and downregulation of the NF-kB and MAPK pathways [28].

## Materials and Methods

### Ethics Statement

The animal study protocol was approved by the Institutional Animal Care and Use Committee of Division of Health Sciences, Graduate School of Medicine, The University of Osaka (approval no. 24–03-03).

### Animal Preparation

A total of 33 mice, 6-week-old, male ddY mice (Japan SLC Ltd., Hamamatsu, Japan) were divided into four groups: (i) sham-instilled group (*n* = 8), (ii) porcine pancreatic elastase (PPE)-treated group (*n* = 6), (iii) EP-treated group (*n* = 12), and (iv) Nic-treated group (*n* = 7). Sham-instilled mice were administered 40 μL of saline intra-tracheally for five consecutive days per week for 21 days. A saline solution of PPE (40 μL, 300 U/kg body weight; FUJIFILM Wako Pure Chemical Corporation, Osaka, Japan) was intra-tracheally administered to each mouse in the PPE-, EP-, and Nic-treated groups to induce lung injury (the day of PPE administration was set as day 0). One day after PPE administration, EP and Nic treatments were initiated in the EP- and Nic-treated groups, respectively, following a similar protocol as previously reported [9]. In brief, a 40 μL saline solution of EP (2.6 mg/kg body weight; Tokyo Chemical Industry Ltd, Tokyo, Japan) or Nic (2.4 mg/kg body weight; BIOMOL International, Pennsylvania, USA) was intra-tracheally administered to each mouse in the EP- or Nic-treated group for five consecutive days per week for a 20-day period. The PPE-treated mice were intra-tracheally administered with 40 μL saline solution for five consecutive days per week for a 20-day period. In all cases, prior to instillation, the mice were anesthetized with 5% isoflurane (ISOFLU®, Dainippon Sumitomo Pharmaceutical Co. Ltd, Osaka, Japan). The survival rate of the 21-day procedure was 100% in all groups.

MRI measurements of sham-instilled mice were conducted on day 0. PPE was administered to each mouse in the EP-, Nic-, and PPE-treated groups on day 0, as described above. One day after PPE administration, MRI measurements were performed for the EP-, Nic-, and PPE-treated groups. During the remaining of the 20-day period, MRI measurements were performed on days 7, 14, and 21 after PPE administration for all groups. A home-built glass mask was attached to the mouth of each mouse to deliver HP ^129^Xe and oxygen and remove exhaust gases. For respiratory-gated imaging, a pulse transducer (AD Instruments Ltd., Dunedin, New Zealand) was positioned on the abdomen of each mouse to synchronize image acquisition with the respiratory motion of spontaneously breathing mice. During MRI measurements, mice were anesthetized with 2% isoflurane, and their body temperature was maintained using warm water circulating through a rubber tube placed on the abdomen. The animal breathing rate was approximately 150 breaths/min. The MR imaging procedure was performed without tracheal intubation or tracheotomy, and hence it was entirely noninvasive.

### MRI Measurements

All MRI measurements were performed using Agilent Unity INOVA 400 WB spectrometer (Agilent Technologies, Inc., Santa Clara, CA, USA) with a 9.4 T vertical magnet (Oxford Instruments Plc., Oxford, UK) and a Highland L-500 Gradient Amp system (Highland Technology, Inc. California, USA). A self-shielded imaging probe with a Litz coil switchable to ^129^Xe and ^1^H frequencies, 32-mm in diameter and 15-mm in length (Clear Bore DSI-1117; Doty Scientific, Inc., Columbia, SC, USA) was used.

High-purity xenon gas (> 99.995%) with ^129^Xe in its natural abundance, 26.4%, mixed with nitrogen (Japan Air Liquide, Tokyo, Japan) was used to produce HP ^129^Xe for MRI measurements using a home-built continuous-flow type apparatus [5–10]. A gas mixture containing HP ^129^Xe and N_2_ (70% HP ^129^Xe and 30% N_2_) was continuously supplied to mice placed in NMR probe at a rate of 50 ml/min through a mask attached to their head. In the mask, O_2_ (Japan Air Liquide, Tokyo, Japan) was mixed with Xe/N_2_ gas mixture at a rate of 12 ml/min immediately prior to inhalation. The mice spontaneously inhaled a 56:24:20 volume mixture of Xe:N_2_:O_2_ gases.

### Assessment of Pulmonary Function

The pulmonary function of gas exchange metric *f*_*D*_ (%), the rate of HP ^129^Xe magnetization diffusing from the gas phase (alveolar air space) to the dissolved phase (alveolar tissue and blood) within a given exchange time, was assessed from HP ^129^Xe MR images acquired using a balanced steady-state free precession (bSSFP) sequence, as described previously [7, 9]. A parametric map of *f*_*D*_ for each mouse was obtained via pixel-by-pixel analysis using MATLAB (MathWorks, Inc., Natick, MA, USA). The map was then averaged to obtain the whole lung *f*_*D*_ value. The measurements were repeated three times, and the obtained *f*_*D*_ values were averaged.

Similarly, the fractional ventilation *r*_*a*_, the alveolar volume fraction of gas turned over in a single breath, was also assessed [7, 9]. A parametric map of *r*_*a*_ and whole lung *r*_*a*_ value were derived using the same process as for *f*_*D*_, and mean *r*_*a*_ values were compared between the groups.

Acquisition parameters of HP ^129^Xe images were as follows: 1000-μs Gaussian-shaped radiofrequency (RF) pulse of flip angle θ = 40º; acquisition bandwidth, 88 kHz; TR/TE = 3.6 ms/1.8 ms; echo train length, 8; number of shots, 4; number of averages, 8; coronal slice thickness, 20 mm; number of slices, 1; matrix, 64 × 32 with a field of view of 80 × 25 mm^2^. Acquisition was commenced after confirming a steady state signal by monitoring ^129^Xe MR spectra obtained by the application of an 8º hard RF pulse with an interval of 2 s.

### Histology

After completion of MRI experiments, the mice were euthanized with a lethal dose of carbon dioxide gas. Lungs were extracted, fixed in 10% formalin, and processed for histological examination by staining with hematoxylin and eosin (H&E), as previously described [7]. Four coronal slices taken close to the center of the lungs per mouse were captured using a digital microscope (Celestron LCD Microscope PRO < CE44345 >; Celestron, LLC., Torrance, CA, USA). The captured images were used to evaluate the mean linear intercept (MLI) and mean bronchial wall thickness (*h*_*bw_histology*_). The MLI and *h*_*bw_histology*_ values were determined from five regions of the lung (right upper lobe, right middle lobe, right lower lobe, and the upper and lower lobes of the left lung). Each set of five values was averaged over the four slides, and the resulting five averaged regional MLI and *h*_*bw*_histology_ values were averaged to yield a single mean MLI and *h*_*bw*_histology_ value for each mouse of the sham-instilled, PPE- and EP-, and Nic-treated groups.

### Statistical Analysis

The *f*_*D*_, *r*_*a*_ and MLI values were compared among the four mouse groups using the Kruskal–Wallis test. When any significant differences were detected, pairwise comparisons were performed using the Mann–Whitney U test. All of the data are presented as mean ± standard error and/or box-and-whisker plots, and differences in signal intensities were considered significant at *P* < 0.05.

## Results

### Temporal Changes in Pulmonary Gas Exchange Function

**Fig. 1:**
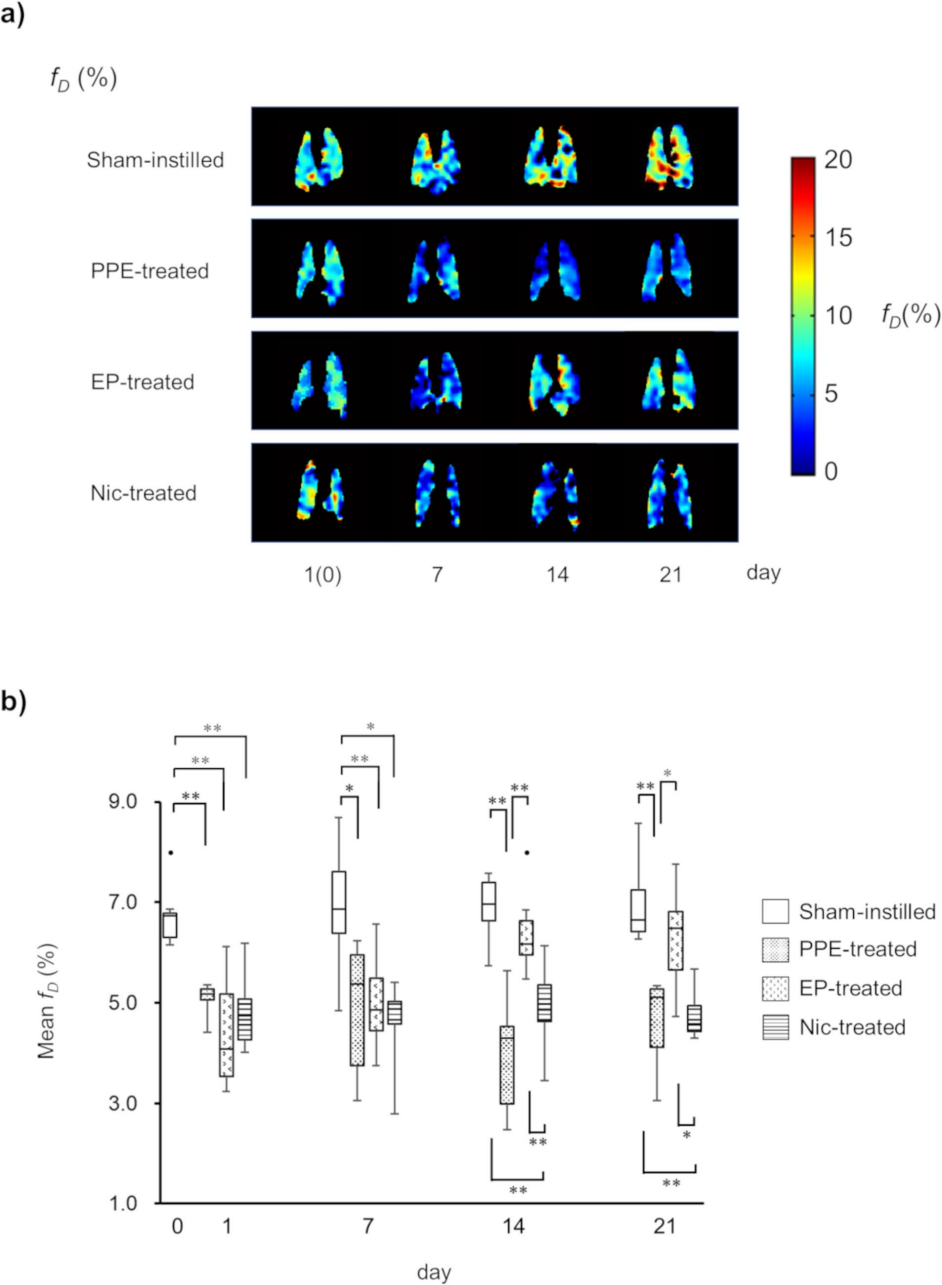
Temporal changes in representative *f*_*D*_ parametric maps of mice of each of the four groups, from top to bottom: sham-instilled; PPE-treated; EP-treated; Nic-treated (**a**). Box plots of temporal changes in the mean *f*_*D*_ values for all mice, separated by groups (**b**). The time course from the initial intra-tracheal injection of saline or PPE is shown horizontally. Significant differences between groups are indicated by solid lines (*: *P* < 0.05, **:*P* < 0.01). Of note, EP and Nic treatments were initiated one day after PPE administration on day 0. The first *f*_*D*_ map of sham-instilled mouse was obtained from day 0, whereas the first maps of PPE-, EP-, and Nic-treated mice were obtained from day 1

Temporal changes in representative *f*_*D*_ parametric maps and *f*_*D*_ values for the sham-instilled, PPE-, EP-, and Nic-treated groups are illustrated in Fig. 1a and 1b, respectively. Notably, EP and Nic treatments were initiated one day after PPE administration (set as day 0). When compared with the *f*_*D*_ of the sham-instilled group on day 0 (*f*_*D*_sham-instilled_ = 6.8 ± 0.7%), significant decrease in the *f*_*D*_ values of the PPE- (*f*_*D*_PPE-treated_ = 5.1 ± 0.4%), EP- (*f*_*D*_EP-treated_ = 4.4 ± 1.2%), and Nic-treated (*f*_*D*_Nic-treated_ = 4.8 ± 0.8%) groups was observed on day 1 after PPE administration (*P* < 0.01). Significant decrease in the *f*_*D*_ value of the PPE-treated group compared with that of the sham-instilled group was consistent for 21 days after PPE administration. Similarly, decrease in the *f*_*D*_ values of both EP- and Nic-treated groups was observed until day 7 after PPE administration. However, the *f*_*D*_ value of the EP-treated group recovered to a similar level as that of the sham-instilled group on day 14 after PPE administration (*f*_*D*_sham-instilled_ = 6.9 ± 0.6% and *f*_*D*_EP-treated_ = 6.6 ± 0.9%), but was significantly higher ​​than that of the PPE and Nic groups (*f*_*D*_PPE-treated_ = 4.0 ± 1.2% and *f*_*D*_Nic-treated_ = 4.9 ± 1.0%, *P* < 0.01). This recovery continued until day 21. However, the *f*_*D*_ value of the Nic group did not recover over the remaining period.

### Temporal Changes in Pulmonary Ventilation Function

**Fig. 2:**
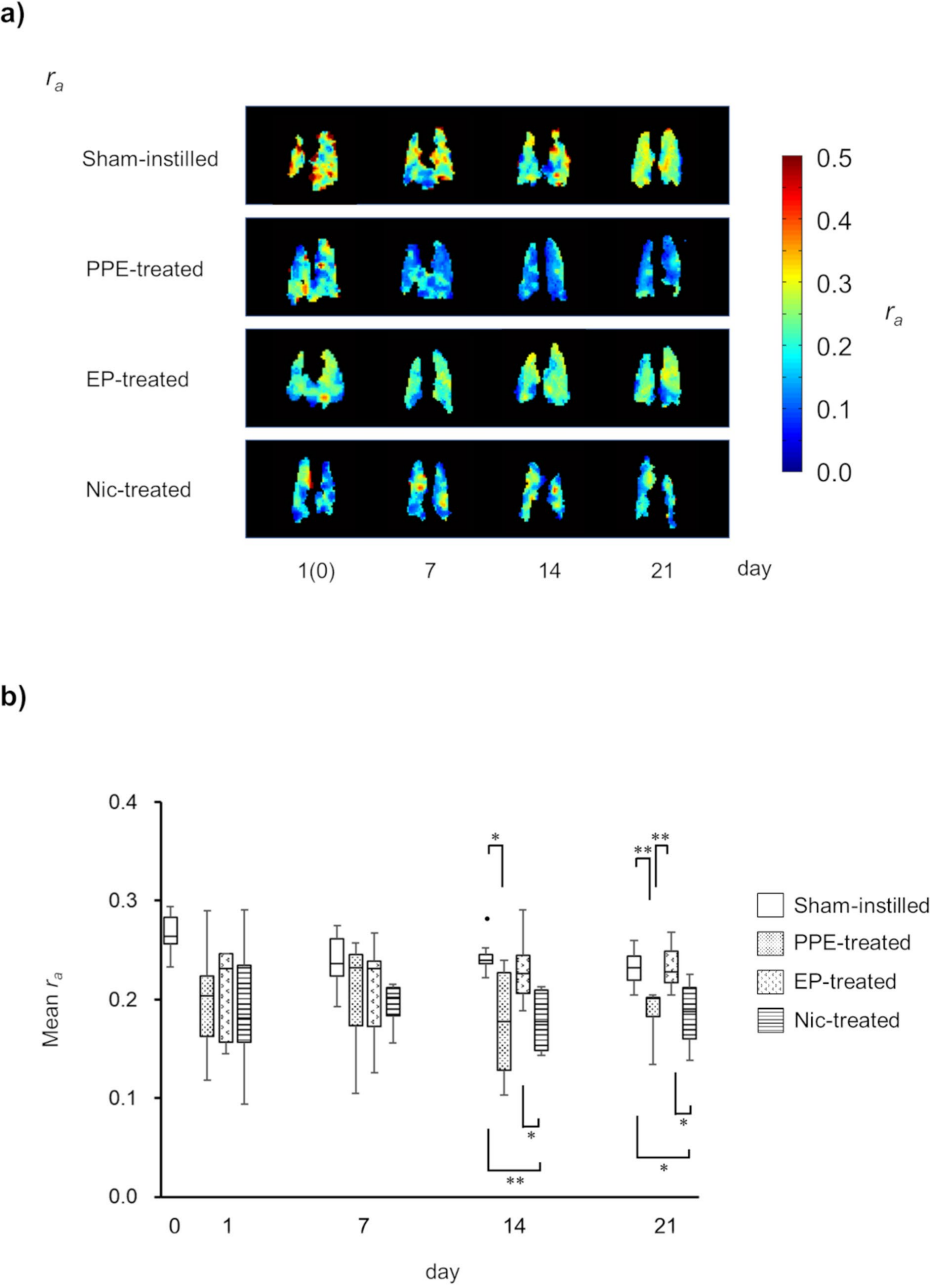
Temporal changes in representative *r*_*a*_ parametric maps of mice of each of the four groups, from top to bottom: sham-instilled; PPE-treated; EP-treated; Nic-treated (**a**). Box plots of temporal changes in the mean *r*_*a*_ values for all mice, separated by groups (**b**)

Temporal changes in the representative *r*_*a*_ parametric maps and *r*_*a*_ values of the sham-instilled, PPE-, EP-, and Nic-treated groups are shown in Fig. 2a and b, respectively. In contrast to the results of *f*_*D*_ measurements, the *r*_*a*_ values of the PPE- (*r*_*a*_PPE-treated_ = 0.20 ± 0.06), EP- (*r*_*a*_EP-treated_ = 0.20 ± 0.05), and Nic-treated (*r*_*a*_Nic-treated_ = 0.19 ± 0.07) groups were not decreased on day 1 after PPE administration compared with the *r*_*a*_ value of the sham-instilled group on day 0 (*r*_*a*_sham-instilled_ = 0.27 ± 0.02). This tendency persisted until day 7. On day 14, the *r*_*a*_ values of the PPE- (*r*_*a*_PPE-treated_ = 0.18 ± 0.06) and Nic-treated (*r*_*a*_Nic-treated_ = 0.17 ± 0.03) groups were significantly decreased compared with that of the sham-instilled group (*r*_*a*_sham-instilled_ = 0.25 ± 0.02, *P* < 0.05). On the other hand, the *r*_*a*_ value of the EP-treated group (*r*_*a*_EP-treated_ = 0.23 ± 0.03) was found to be significantly higher than that of the Nic-treated group on day 14 (*P* < 0.05). On day 21, the *r*_*a*_ value of the EP-treated group (*r*_*a*_EP-treated_ = 0.23 ± 0.02, *P* < 0.01) was significantly increased when compared with that of the sham-instilled group(*r*_*a*_sham-instilled_ = 0.23 ± 0.02, *P* < 0.01) and that of the Nic-treated group (*r*_*a*_Nic-treated_ = 0.19 ± 0.03).

### Histograms of Pulmonary Gas Exchange Function

**Fig. 3:**
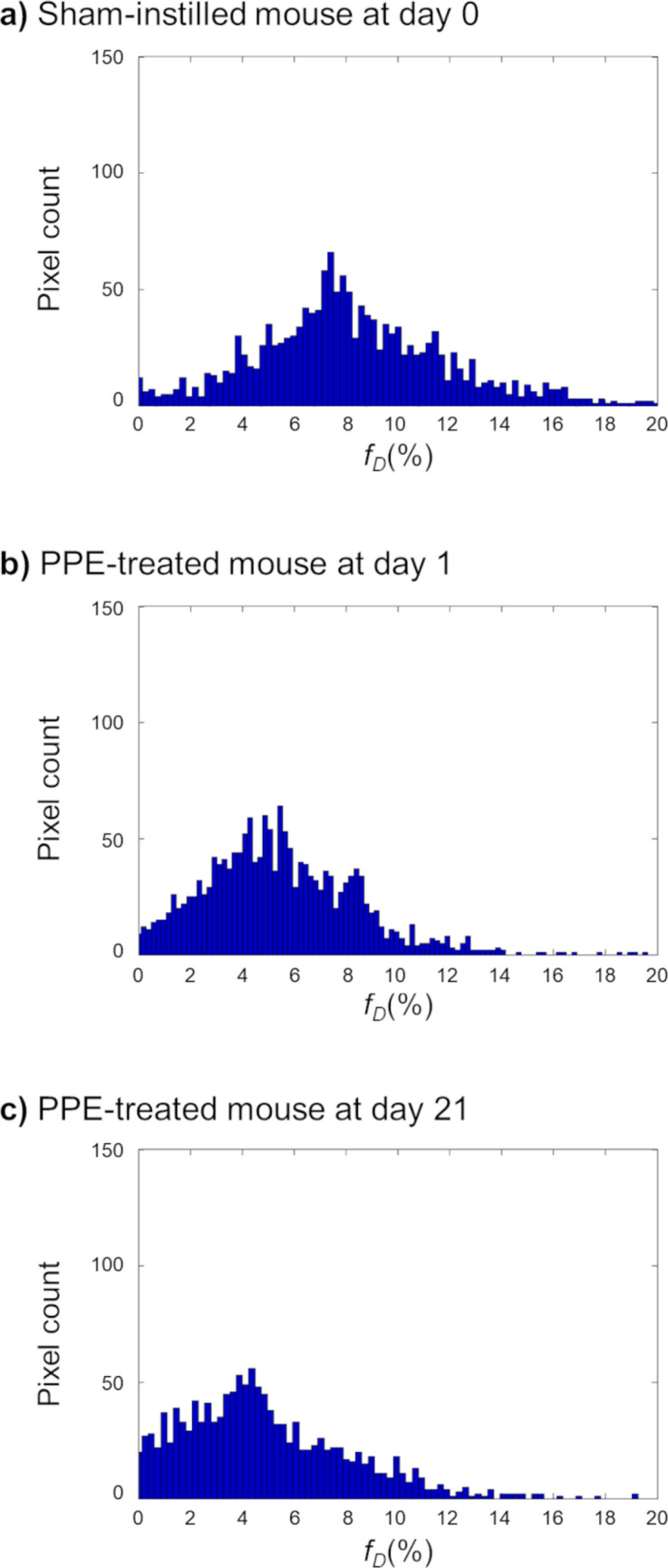
Representative histograms of *f*_*D*_ values obtained from sham-instilled mice (**a**), PPE-treated mice on day 1 (**b**), and PPE-treated mice on day 21 (**c**). The global mean *f*_*D*_ = 7.2% (**a**), 5.1% (**b**), and 4.9% (**c**). After PPE injection, *f*_*D*_ values tended to shift ​​to lower values ​​without a decrease in the total area of ​​the histogram, suggesting regional transfer from higher *f*_*D*_ sites to lower *f*_*D*_ sites as a result of the pathological process

Figure 3 shows the comparative results of representative histograms of *f*_*D*_ values obtained from *f*_*D*_ maps of a sham-instilled mouse on day 0, a PPE-treated mouse on day 1, and a PPE-treated mouse on day 21.

### Histology

**Fig. 4:**
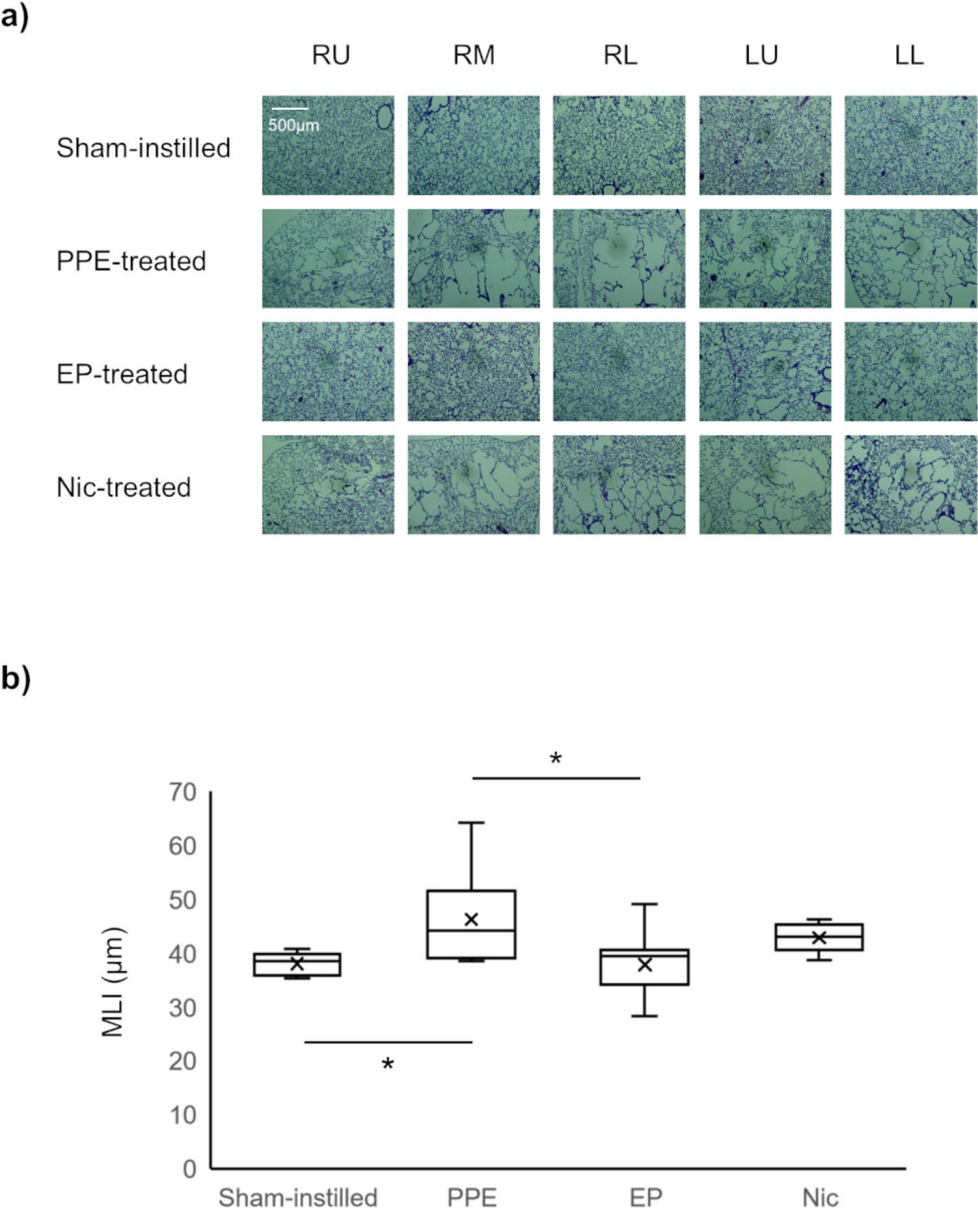
Representative H&E-stained histology slides obtained from five lung regions of a mouse from each of the four groups, from top to bottom: sham-instilled; PPE-treated; EP-treated; Nic-treated (**a**). *RU*, right upper lobe; *RM*, right middle lobe; *RL*, right lower lobe; *LU*, upper region of the left lobe; *LL*, lower region of the left lobe. Box plots of mean MLI values obtained from mice of each of the four groups (**b**)

Representative histological images obtained from mice of the sham-instilled, PPE-, EP-, and Nic-treated groups are shown in Fig. 4a. The whole lung mean MLI values obtained from the sham-instilled, PPE-, EP-, and Nic-treated groups are shown in Fig. 4b. As illustrated in Fig. 4a, alveolar enlargement was observed in the PPE- and Nic-treated groups. The mean MLI of the PPE-treated group (*MLI*_*PPE-treated*_ = 46.3 ± 9.4 μm) was significantly higher than that of the sham-instilled and EP-treated groups (*MLI*_*Sham-instilled*_ = 38.0 ± 2.0 μm and *MLI*_*EP-treated*_ = 37.8 ± 5.6 μm, *P* < 0.05). The MLI of the Nic-treated group (*MLI*_*Nic-treated*_ = 42.8 ± 2.7 μm) was not significantly different from that of the sham-instilled, PPE-, and EP-treated groups.

**Fig. 5:**
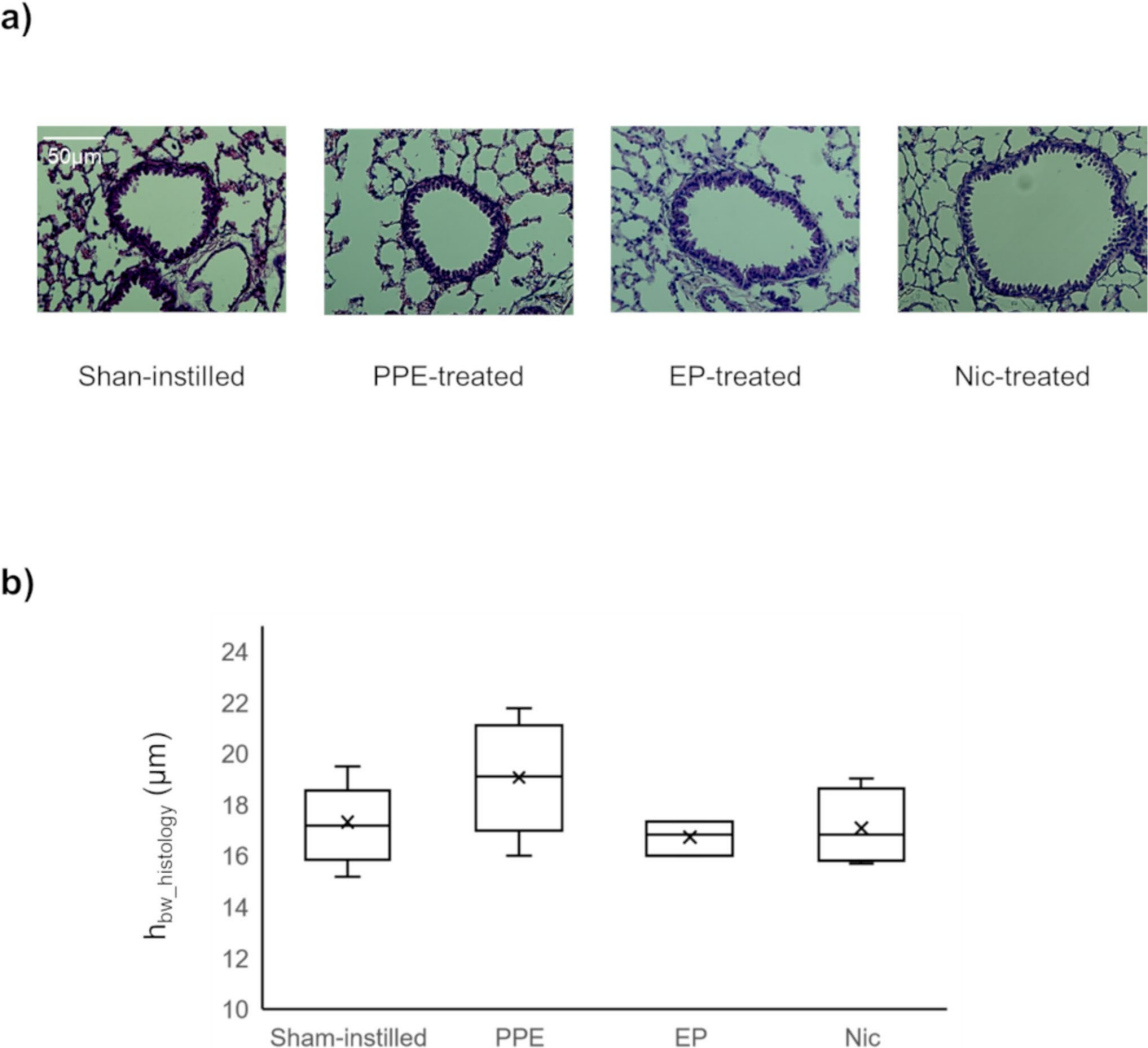
Representative H&E-stained histology slides obtained from the four groups, from left to right: sham-instilled; PPE-treated, EP-treated; Nic-treated (**a**). Box plots showing the mean bronchial wall thickness (*h*_*bw_histology*_) values obtained from mice of each of the four groups

Representative histological images and whole lung mean *h*_*bW_histology*_ values obtained from mice of the sham-instilled, PPE-, EP-, and Nic-treated groups are shown in Fig. 5. There were no significant differences in *h*_*bW_histology*_ values between the groups.

### Mechanisms of Therapeutic Effect of EP and Nic

**Fig. 6:**
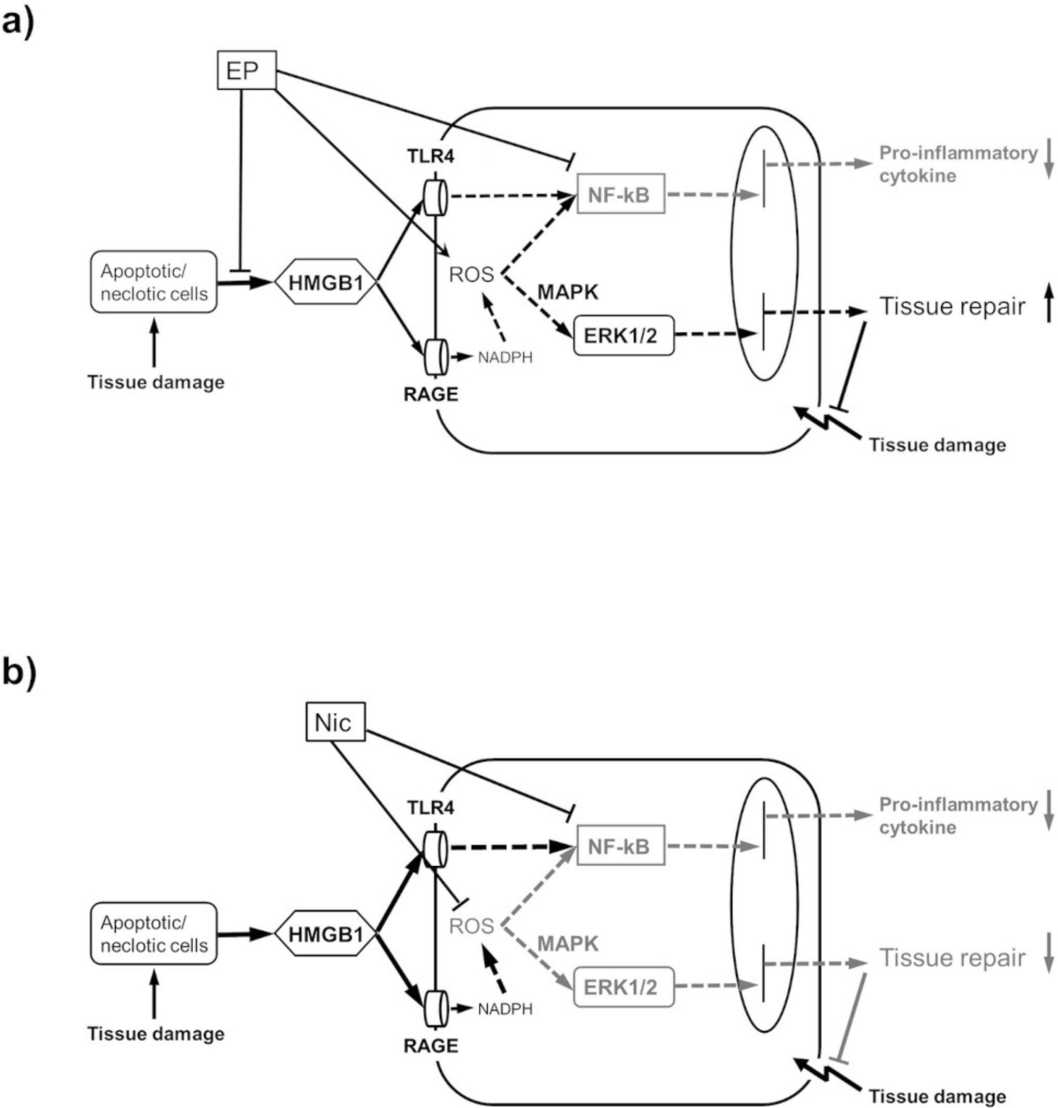
Proposed mechanisms of therapeutic effect of EP against elastase-induced lung injury. **a**) EP downregulates HMGB1 expression and moderately activates intracellular ROS and MAPK/ERK pathway, thereby initiating wound healing process. **b**) Nic, which inhibits intracellular ROS and deactivates the MAPK/ERK pathway, does not exhibit a positive therapeutic effect

Figure 6 depicts the mechanisms underlying the therapeutic effect of EP (Fig. 6a) and Nic (Fig. 6b) against the elastase-induced lung injury proposed in this study.

## Discussion

In the present study, we successfully monitored the temporal changes in pulmonary function, *f*_*D*_ and *r*_*a*_, induced by PPE administration using HP ^129^Xe MRI (Figs. 1 and 2). One day after PPE administration, the *f*_*D*_ values of the PPE-, EP-, and Nic-treated groups were significantly lower than that of the sham-instilled group on day 0 (Fig. 1). Emphysema was previously reported to develop seven days after PPE administration [29]. Therefore, our data suggest that the *f*_*D*_ metric can detect changes in pulmonary function due to pathology before emphysema development. This observation is similar to that of our previous study using a mouse model of lung cancer, in which inflammation-induced alveolar septal wall thickening reduced *f*_*D*_ values prior to the onset of lung cancer [10]. On the other hand, one day after PPE administration, no significant changes were observed in the *r*_*a*_ values of the PPE-, EP-, and Nic-treated groups compared with that of the sham-instilled group on day 0 (Fig. 2). In our previous study, we reported that inflammation-induced bronchial wall thickening reduced *r*_*a*_ values [7]. In the present study, bronchial wall thickening was not observed in any histological images of the PPE-, EP-, or Nic-treated groups compared with those of the sham-instilled group (Fig. 5), suggesting no changes in *r*_*a*_ values were observed one 1 day after PPE administration.

Considering the above observations, although no alveolar septal wall thickening was observed histologically 21 days after PPE administration (Fig. 4), we assumed that alveolar septal wall thickening might occur without bronchial wall thickening due to inflammation on day 1 after PPE administration, before the onset of emphysema. Previously, using a mouse model of elastase-induced lung injury, we reported temporal changes in alveolar septal wall thickness *in vivo* (Figure E5 of Ref. [29]). In this study, significant thickening of the alveolar septal walls was observed *in vivo* on day 1 after PPE administration, prior to the development of emphysema on day 7. In addition, no alveolar septal wall thickening was observed *in vivo* on day 7 after PPE administration. This study supports these assumptions. Notably, in addition to inflammation, a reduction in alveolar tissue volume was reported one day after PPE administration [29]. This finding suggests that, in the present study, tissue destruction to some extent might have already started on day 1 after PPE administration, which is supported by shifts in *f*_*D*_ values caused by PPE administration (Fig. 3). When compared with the *f*_*D*_ values of a sham-instilled mouse on day 0, the *f*_*D*_ values of a PPE-treated mouse on day 1 showed a wider distribution and tended to shift to lower values, similar to that of a PPE-treated mouse on day 21. Therefore, to observe therapeutic effects, it is rational to initiate EP and Nic treatment one day after PPE administration as a significant decrease in *f*_*D*_ value was observed at this time point in the present study.

The decrease in *f*_*D*_ value of the PPE-treated group was consistent from day 1 to day 21 after PPE administration (Fig. 1). As mentioned above, emphysema develops seven days after PPE administration [29]. Therefore, the decrease in *f*_*D*_ value on day 7 after PPE administration was due to the early onset of emphysematous lesions. In contrast, the *r*_*a*_ value of the PPE-treated group was significantly lower than that of the sham-instilled group on day 14 after PPE administration (Fig. 2), implying that emphysematous lesions fully developed at this time point. In contrast, the *r*_*a*_ value of the EP-treated group was higher than that of the Nic-treated groups on day 14 after PPE administration and recovered to a level similar to that of the sham-instilled group, similar to that of *f*_*D*_ value (Figs. 1 and 2). The recovery of both *f*_*D*_ and *r*_*a*_ continued until day 21 after PPE administration, and the MLI of the EP-treated group was significantly lower than that of the PPE-treated group (Fig. 4). Therefore, these data suggest that by starting EP treatment one day after PPE administration, decline in pulmonary function associated with the onset of emphysematous lesions could be improved, as measured by HP ^129^Xe MRI. However, in the Nic-treated group, the decline in the *r*_*a*_ and *f*_*D*_ values did not improve (Figs. 1 and 2). Moreover, the MLI of the Nic-treated group did not improve compared with that of the PPE-treated group (Fig. 4), supporting the results of the pulmonary functional assessments.

Regulation of the MAPK/ERK pathway has been reported to promote tissue repair during cutaneous wound healing [20]. The MAPK/ERK pathway has been reported to be activated during wound healing in a murine diabetic wound model [21]. Regulation of HMGB1 expression has been reported to be involved in wound healing process via the MAPK/ERK pathway [22]. Based on these reports, we hypothesize that EP regulates the expression levels of HMGB1 and the MAPK/ERK pathway to exert anti-inflammatory and tissue repair effects. Our previous study using a mouse model of bleomycin-induced lung injury supported this hypothesis, in which we observed lung structural and functional changes caused by the upregulation and downregulation of HMGB1 expression [9]. However, in this study, we were unable to directly elucidate the involvement of intracellular ROS and MAPK/ERK pathway in the recovery of injured tissue.

In the present study, we investigated the involvement of intracellular ROS and MAPK/ERK pathway in elastase-induced emphysema in mice by evaluating and comparing the pharmacological effects of EP and Nic. Intracellular ROS production has been reported to contribute to elastase-induced lung injury [30]. Further, HMGB1 and NF-kB have also been reported to be involved in elastase-induced lung injury [31, 32]. Furthermore, EP activated the ROS-MAPK/ERK pathway to inhibit melanogenesis in B16F10 melanoma cells [27]. Therefore, we hypothesized that EP moderately upregulates the expression levels of HMGB1 and activates the ROS-MAPK/ERK pathway to exert anti-inflammatory and tissue repair effects (Fig. 6a). In contrast, Nic suppressed ROS production and showed its protective effect through downregulation of the NF-kB and MAPK pathways (Fig. 6b) [28]. In the present study, Nic treatment did not improve pulmonary function, indicating the role of the MAPK/ERK pathway in wound healing process. In other words, non-activation of the MAPK/ERK pathway by Nic treatment seemed to impair tissue repair and lead to the development of emphysema and pulmonary functional decline.

Although Nic has been reported to show protective effects against acute lung injury [28], collapse-induced lung injury [33], pulmonary fibrosis [34, 35], pulmonary artery endothelial damage in pulmonary hypertension [36], and other lung disorders [37], its therapeutic effects have not been reported. In the present study, inflammation was detected one day after PPE administration, and Nic treatment was started at this stage to suppress the inflammation through inhibition of intracellular ROS production and NF-kB activation [28]. However, at this stage, in addition to inflammation, there appeared to be some extent of tissue destruction, as mentioned previously. We assumed that Nic did not show tissue repair effects via the MAPK/ERK pathway and was unable to exert a therapeutic effect considering the above reason. In contrast, EP downregulated HMGB1 expression, increased ROS production, and moderately upregulated the MAPK/ERK pathway (Fig. 6a) [24–27], resulting in tissue repair and suppression in the onset of emphysema, leading to improvement in pulmonary function.

Finally, owing to the limited sample size of this study, the observed associations between lung function parameters (*f*_*D*_ and *r*_*a*_) obtained from HP ^129^Xe MRI and the treatment effects of EP and Nic should be interpreted with caution. Further validation using a larger cohort is required to confirm these findings.

## Conclusions

In the present study, we successfully monitored temporal changes in pulmonary function of gas exchange and ventilation caused by the development of emphysema using a preclinical HP ^129^Xe MRI protocol. The treatment responses of EP and Nic against the development of emphysema were monitored and compared. EP treatment effectively repaired tissue damage and improved pulmonary function in elastase-induced lung injury, whereas Nic showed no therapeutic effects. The present results suggest therapeutic efficacy of EP in wound healing process via activation of the MAPK/ERK pathway.

## Supplementary Information

Below is the link to the electronic supplementary material.

ESM 1 DOCX (363 KB)

## Data Availability

The datasets generated in this study are available from the corresponding author upon reasonable request.
