# Supplementary material for: Ethyl Pyruvate Promotes Wound Healing in Elastase-Induced Lung Injury in Mice as Assessed by Hyperpolarized 129Xe Magnetic Resonance Imaging
Source: Mol Imaging Biol. 2025 Dec 10;28(1):169–80. doi: 10.1007/s11307-025-02073-6 (PMC12966195; doi:10.1007/s11307-025-02073-6)
Supplement: Supplementary file 1 — DOCX (363 KB) [file 11307_2025_2073_MOESM1_ESM.docx]

**Electronic Supplementary Material**

**Ethyl Pyruvate Promotes Wound Healing in Elastase-Induced Lung Injury in Mice as Assessed by Hyperpolarized ^129^Xe Magnetic Resonance Imaging**

**Journal: Molecular Imaging and Biology**

Atsuomi Kimura^1^ · Akihiro Shimokawa^1^ · Neil J. Stewart^2^ · Rie Hosoi^1^ · Hirohiko Imai^3^ · Hideaki Fujiwara^1^

^1^ Division of Health Sciences, Graduate School of Medicine, The University of Osaka, 1-7 Yamadaoka, Suita, Osaka 565-0871, Japan

^2^ POLARIS, Division of Clinical Medicine, School of Medicine & Population Health, Faculty of Health, University of Sheffield, Sheffield, United Kingdom

^3^ Innovation Research Center for Quantum Medicine, Gifu University School of Medicine, 1-1 Yanagido, Gifu 501-1194, Japan

**Corresponding author:**

Atsuomi Kimura

Suita, Osaka, Japan, 565-0871

Tel: +81-6-6879-2578

Email: [kimura@sahs.med.osaka-u.ac.jp](mailto:kimura@sahs.med.osaka-u.ac.jp)

**Materials and Methods**

**MRI Measurements**

Respiratory-gated imaging of the mouse lung was performed according to the method described in our previous report [1].

**Assessment of Pulmonary Function**

The methodology for assessing pulmonary function using the gas exchange metric, *f_D_* (%), is based on the xenon polarization transfer contrast method [2]. Similarly, the fractional ventilation *r_a_* was assessed using the method described in our previous report [3].

In the acquired HP ^129^Xe images, appropriate thresholding was applied in order to differentiate the lung tissue from the background and exclude low signal-to-noise ratio (SNR) regions near the lung periphery from the parametric map calculations. The appropriate threshold level was determined by confirming that the entire lung parenchyma was preserved following masking of low-intensity pixels. Pixels with image intensities of less than 30% of the maximum pixel value were excluded from the performed analysis.

Acquisition parameters for HP ^129^Xe MR using the bSSFP sequence were determined as previously reported [4].

**Results**

The HP ^129^Xe MRI-derived parameters of pulmonary function (*f_D_* and *r_a_*) during the 21-day experimental protocol obtained from the four mice groups are listed in Tables 1 and 2.

Representative spatial distributions of high *f_D_* and *r_a_* values obtained from two sham-instilled mice are shown in Supplementary Figure 1.

The relationships between the *f_D_* and *r_a_* values on day 21 and histology-derived parameters of lung structure (MLI) obtained from the four mice groups are shown in Supplementary Figure 2.

**Supplementary Table 1.** Experimentally determined metrics of *f_D_* of the four mice groups^*^

|  |  |  |  | Day |  |  |  |
| --- | --- | --- | --- | --- | --- | --- | --- |
|  | mouse | 0 | 1 | 7 | 14 | 21 |  |
|  | Sham-instilled | 6.8 (0.7) |  | 6.9 (1.2) | 6.9 (0.6) | 7.0 (0.8) |  |
|  | PPE-treated |  | 5.1 (0.4) | 4.9 (1.4) | 4.0 (1.2) | 4.6 (1.0) |  |
|  | EP-treated |  | 4.4 (1.2) | 5.0 (0.8) | 6.6 (0.9) | 6.3 (0.9) |  |
|  | Nic-treated |  | 4.8 (0.8) | 4.5 (1.0) | 4.9 (1.0) | 4.8 (0.9) |  |

***** Values of *f_D_* represent the mean ± SD.

**Supplementary Table 2.** Experimentally determined metrics of *r_a_* of the four mice groups^*^

|  |  |  |  | Day |  |  |  |
| --- | --- | --- | --- | --- | --- | --- | --- |
|  | mouse | 0 | 1 | 7 | 14 | 21 |  |
|  | Sham-instilled | 0.27 (0.02) |  | 0.24 (0.03) | 0.25 (0.02) | 0.23 (0.02) |  |
|  | PPE-treated |  | 0.20 (0.06) | 0.20 (0.06) | 0.18 (0.06) | 0.18 (0.03) |  |
|  | EP-treated |  | 0.20 (0.05) | 0.21 (0.05) | 0.23 (0.03) | 0.23 (0.02) |  |
|  | Nic-treated |  | 0.19 (0.07) | 0.19 (0.03) | 0.17 (0.03) | 0.19 (0.03) |  |

***** Values of *r_a_* represent the mean ± SD.


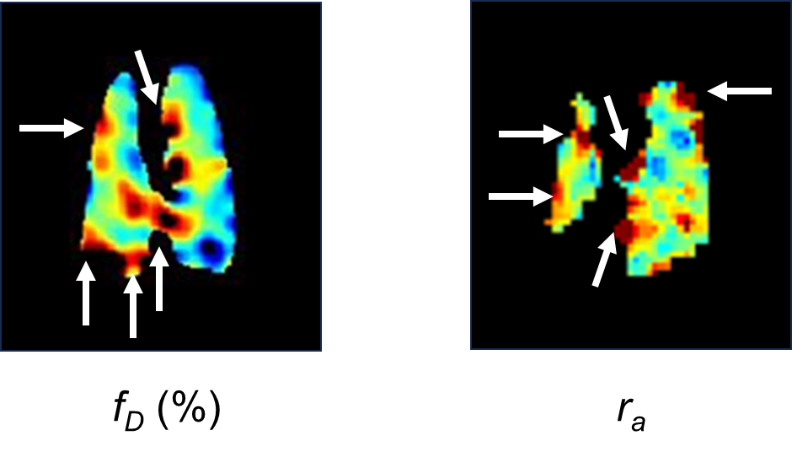


**Supplementary Figure 1.** Representative spatial distributions of high *f_D_* and *r_a_* values obtained from two sham-instilled mice. Arrows indicate the elevated values.


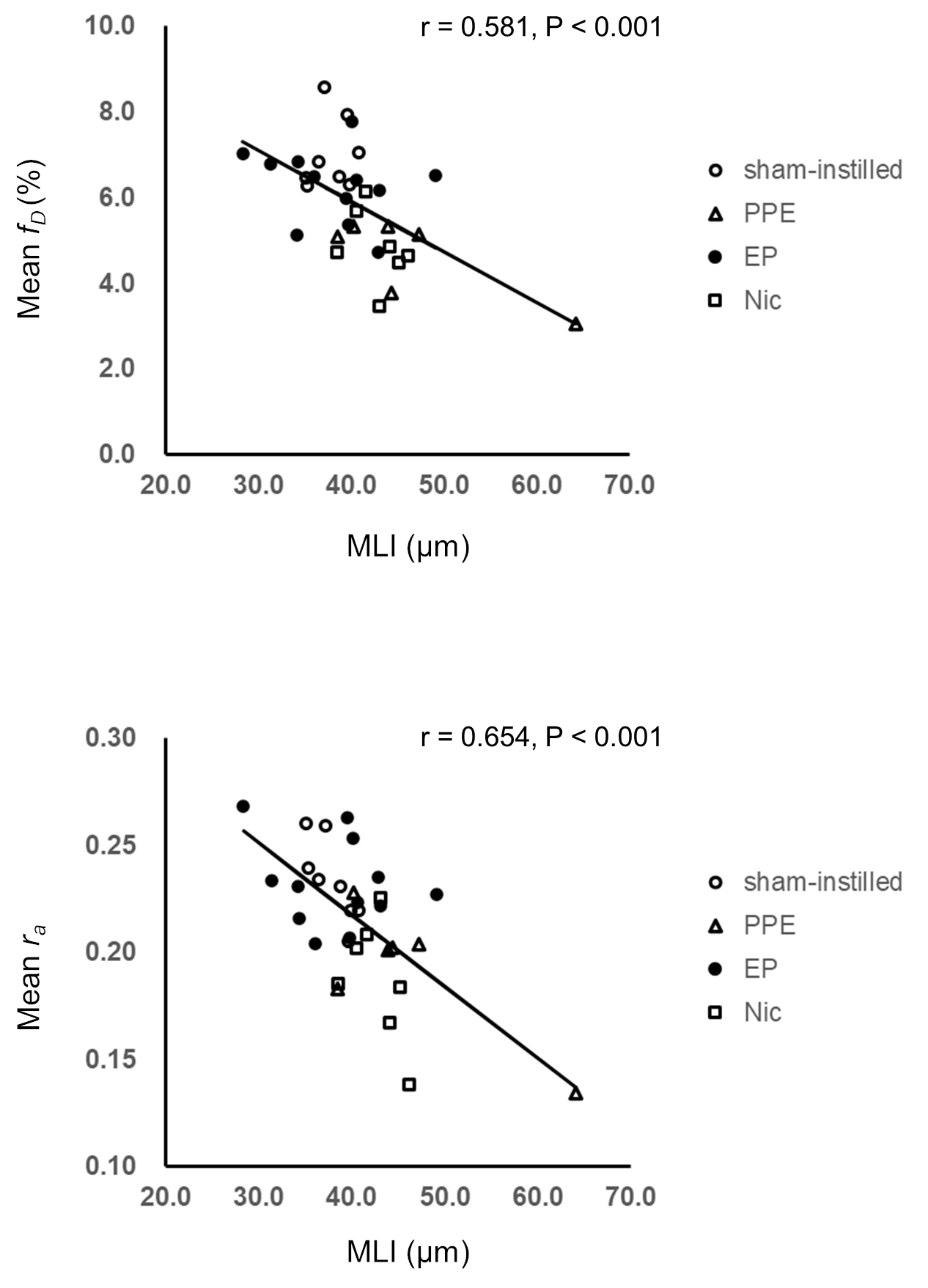


**Supplementary Figure 2.** Relationships between *f_D_*, *r_a_*, and MLI obtained from the sham-instilled (○), PPE-treated (▲), EP-treated (●), and Nic-treated (□) mice after the 21-day experimental protocol. The Pearson’s *r* value and *P* value of statistical significance are shown in each plot.

**Limitations**

For the MRI measurements, the number of averages was 8. Occasionally, elevated *f_D_* and *r_a_* values were predominantly observed at the lung boundaries (Supplementary Figure 1, white arrows). This phenomenon may reflect a volume mismatch between averages. Despite the use of respiratory-gated imaging, capturing identical lung morphology under spontaneous breathing conditions remained challenging. Although the mismatch-affected region represents only a limited portion of the lung, as shown in the maps, such mismatch is likely a common issue in quantitative assessments using parametric maps—even in studies employing breath-hold techniques with a ventilator. However, in this study, the mismatch is unlikely to affect the accuracy of the measurements, although it may influence data precision. This is supported by the *r_a_* measurement results. Whole-lung *r_a_* values can be approximately compared to global measures of tidal volume (TV) and functional residual capacity (FRC) using the relationship: *r_a_* = TV/(FRC + TV). An *r_a_* value of 0.21 is estimated based on a TV of 0.1 mL and a FRC of 0.38 mL, as measured by micro-CT in spontaneously breathing healthy mice [5]. In the present study using HP ^129^Xe in healthy mice, a reasonable *r_a_* value of approximately 0.25 with good reproducibility was obtained. This consideration is further supported by the observed correlations between *f_D_*, *r_a_*, and the MLI, as shown in Supplementary Figure 2.

For the MRI measurements, we acquired two-dimensional (2D) images of the lungs and evaluated lung function based on these images. The *f_D_* and *r_a_* maps of PPE-, EP-, and Nic-treated mice revealed a relatively uniform decline and recovery in lung function, which made local assessment challenging. In future studies, although it is more time-consuming, it will be necessary to acquire three-dimensional (3D) images and then compare them with the pathological histology of the entire lung. Furthermore, to characterize the distributional features and assess the structural differences between groups, systematic evaluation of the histograms of *f_D_* and *r_a_* using statistical parameters, such as kurtosis and skewness, is required.

Finally, owing to the limited sample size of this study, the observed associations between lung function parameters (*f_D_* and *r_a_*) obtained from HP ^129^Xe MRI and the treatment effects of EP and Nic should be interpreted with caution. Further validation using a larger cohort is required to confirm these findings.

**References**

1. Imai H, Kimura A, Hori Y, et al. (2011) Hyperpolarized 129Xe lung MRI in spontaneously breathing mice with respiratory gated fast imaging and its application to pulmonary functional imaging. NMR Biomed 24:1343-1352
2. Ruppert K, Brookeman JR, Hagspiel KD, Mugler JP, III. (2000) Probing lung physiology with xenon polarization transfer contrast (XTC). Magn Reson Med 44:349-357
3. Imai H, Matsumoto H, Miyakoshi E, et al. (2015) Regional fractional ventilation mapping in spontaneously breathing mice using hyperpolarized ¹²⁹Xe MRI. NMR Biomed 28:24-29
4. Imai F, Kashiwagi R, Imai H, et al. (2011) Hyperpolarized 129Xe MR imaging with balanced steady-state free precession in spontaneously breathing mouse lungs. Magn Reson Med Sci 10:33-40
5. Ford NL, Ren X, Egoriti L, et al. (2025) Respiratory-gated micro-computed tomography imaging to measure radiation-induced lung injuries in mice following ultra-high dose-rate and conventional dose-rate radiation therapy. J Med Imaging (Bellingham) 12:014002
